# Association between lipid-A-producing oral bacteria of different potency and fractional exhaled nitric oxide in a Norwegian population-based adult cohort

**DOI:** 10.1186/s12967-023-04199-z

**Published:** 2023-05-29

**Authors:** Maryia Khomich, Huang Lin, Andrei Malinovschi, Susanne Brix, Lucia Cestelli, Shyamal Peddada, Ane Johannessen, Carsten Eriksen, Francisco Gomez Real, Cecilie Svanes, Randi Jacobsen Bertelsen

**Affiliations:** 1 Department of Clinical Science, University of Bergen, Bergen, Norway; 2 Biostatistics and Computational Biology Branch, National Institute of Environmental Health Sciences (NIEHS), NIH, Research Triangle Park, Durham, NC USA; 3 Department of Medical Sciences, Clinical Physiology, Uppsala University, Uppsala, Sweden; 4 Department of Biotechnology and Biomedicine, Technical University of Denmark, Kongens Lyngby, Denmark; 5 Department of Global Public Health and Primary Care, Center for International Health, University of Bergen, Bergen, Norway; 6 Center for Molecular Prediction of Inflammatory Bowel Disease (PREDICT), Department of Clinical Medicine, Aalborg University, Copenhagen, Denmark; 7 Department of Obstetrics and Gynecology, Haukeland University Hospital, Bergen, Norway; 8 Department of Occupational Medicine, Haukeland University Hospital, Bergen, Norway; 9 Oral Health Center of Expertise in Western Norway, Bergen, Norway

**Keywords:** Lipopolysaccharide, Endotoxin, Lipid A, Oral microbiota, Bacteria, Airway inflammation, Exhaled nitric oxide, RHINESSA, MiRKAT, ANCOM-BC

## Abstract

**Background:**

Lipid A is the primary immunostimulatory part of the lipopolysaccharide (LPS) molecule. The inflammatory response of LPS varies and depends upon the number of acyl chains and phosphate groups in lipid A which is specific for a bacterial species or strain. Traditional LPS quantification assays cannot distinguish between the acylation degree of lipid A molecules, and therefore little is known about how bacteria with different inflammation-inducing potencies affect fractional exhaled nitric oxide (F_eNO_). We aimed to explore the association between pro-inflammatory hexa- and less inflammatory penta-acylated LPS-producing oral bacteria and F_eNO_ as a marker of airway inflammation.

**Methods:**

We used data from a population-based adult cohort from Norway (*n* = 477), a study center of the RHINESSA multi-center generation study. We applied statistical methods on the bacterial community- (prediction with MiRKAT) and genus-level (differential abundance analysis with ANCOM-BC) to investigate the association between the oral microbiota composition and F_eNO_.

**Results:**

We found the overall composition to be significantly associated with increasing F_eNO_ levels independent of covariate adjustment, and abundances of 27 bacterial genera to differ in individuals with high F_eNO_ vs. low F_eNO_ levels. Hexa- and penta-acylated LPS producers made up 2.4% and 40.8% of the oral bacterial genera, respectively. The Bray–Curtis dissimilarity within hexa- and penta-acylated LPS-producing oral bacteria was associated with increasing F_eNO_ levels independent of covariate adjustment. A few single penta-acylated LPS producers were more abundant in individuals with low F_eNO_ vs. high F_eNO_, while hexa-acylated LPS producers were found not to be enriched.

**Conclusions:**

In a population-based adult cohort, F_eNO_ was observed to be associated with the overall oral bacterial community composition. The effect of hexa- and penta-acylated LPS-producing oral bacteria was overall significant when focusing on Bray–Curtis dissimilarity within each of the two communities and F_eNO_ levels, but only penta-acylated LPS producers appeared to be reduced or absent in individuals with high F_eNO_. It is likely that the pro-inflammatory effect of hexa-acylated LPS producers is counteracted by the dominance of the more abundant penta-acylated LPS producers in this population-based adult cohort involving mainly healthy individuals.

**Supplementary Information:**

The online version contains supplementary material available at 10.1186/s12967-023-04199-z.

## Background

Lipopolysaccharide (LPS) is a major cell wall component of Gram-negative bacteria, and a strong stimulator of innate immunity in eukaryotes [1, 2]. LPS, also called endotoxin, is structurally heterogeneous and consists of three distinct domains: highly conserved lipid A, core oligosaccharide, and a highly variable O-antigen, while a few bacteria, e.g., *Porphyromonas gingivalis,* contain LPS molecules composed only of lipid A [3–5]. Lipid A is the primary immunostimulatory part of the LPS molecule, and it mainly activates innate pathways in macrophages, monocytes, and monocyte-derived dendritic cells that all express Toll-like receptor 4 (TLR4) [3, 6–8]. LPS is first recognized by myeloid differentiation factor 2 (MD-2) which binds five of the acyl chains of LPS (penta-acylated LPS) before it assembles with TLR4 in the TLR4-MD2 complex. If the lipid A holds a sixth acyl chain (hexa-acylated LPS), it can facilitate the formation of an MD-2-TLR4-TLR4-dimer that finally activates the TLR4 pathway. If lipid A exists in a penta-acylated form, it cannot form a potent activating MD-2-TLR4-TLR4-dimer. The inflammatory response induced by LPS depends upon the structural acyl variations of the lipid A domain [3, 9, 10], but also the number of phosphate groups [11]. The ability of a bacterium to produce LPS variants is encoded in its genome via the presence or absence of the genes encoding the nine enzymes taking part in the Raetz pathway [12]. Most of the Gram-negative bacteria encode the genes for eight of these enzymes ending with the *LpxL* gene (Enzyme Commission (EC) 23.1.241) required for the production of penta-acylated LPS, while only a few bacteria hold the additional *LpxM* gene (EC 23.1.243) required for the production of hexa-acylated LPS, such as found in *E.* *coli* [5]. Once genomes are available, it is, therefore, possible to use in silico prediction based on the presence/absence of bacterial genes to predict their ability for penta-acylated vs. hexa-acylated LPS production. Hexa-acylated LPS is 100 times more potent in stimulating inflammatory response than penta-acylated LPS; in contrast, lipid A with four acyl chains (tetra-acylated LPS) acts as a TLR4 antagonist by dampening the inflammatory response in the host [8, 13]. The ratio between different lipid A variants originating from different bacterial species determines the inflammatory potential [14]. Notably, to evade immune detection, certain bacteria, such as the human pathogenic *Yersinia* species*,* can express hexa- or tetra-acylated LPS forms depending upon environmental conditions [3, 9, 10], or produce a mix of LPS variants, e.g., periodontopathogen *P.* *gingivalis* [15]. Hence, the variation in expressed lipid A forms across bacterial species in a sample makes it challenging to detect and quantify their production [16].

Endotoxin is ubiquitous in the environment and can be present in ambient air particulate matter, contaminated water, house dust, and in occupational settings, such as cotton, saw or grain dust [17]. The oral route is the main gateway for commensal and pro-inflammatory bacteria and their metabolites to enter the lungs [18]. Circulating LPS leads to low-grade inflammation locally (e.g. oral cavity) or systemically (e.g. small intestine), and endotoxin exposure has been associated with increased risk for periodontitis [19, 20], cardiovascular diseases [21], and respiratory diseases, such as occupational lung disease [17] and asthma [22]. Early-life exposure to hexa- and penta-acylated LPS—from farming and livestock sources—is suggested to be protective against allergy and asthma development in adulthood [23, 24].

Traditional LPS quantification assays, such as the Limulus Amebocyte Lysate (LAL) assay, cannot distinguish between different lipid A variants and LPS producers, and little is known about how bacteria with different inflammation-inducing potential affect fractional exhaled nitric oxide (F_eNO_). Exhaled nitric oxide (NO) is a marker of airway inflammation that is used to support asthma diagnosis [25] and guide treatment [26]. Exhaled nitric oxide is believed to reflect type 2 airway inflammation in asthma [26]. In healthy individuals, another relevant source of nitric oxide might be the contribution from the oral cavity by reduction of nitrate (NO_3_^−^) to nitrite (NO_2_^−^) and further reduction to nitric oxide [27]. For example, nitrate load has resulted in increased levels of F_eNO_ and this is different between individuals [28]. Moreover, other mechanisms, e.g., occupational endotoxin exposure, may result in increased levels of F_eNO_ in non-smoking, non-atopic adults [29], probably by means of nitric oxide synthase (NOS) activation in response to the release of inflammatory cytokines such as tumor necrosis factor-α (TNF-α) [30].

Here, we used amplicon sequencing of the oral microbiota and F_eNO_ measurements to investigate the association between the oral microbiota and airway inflammation in a population-based adult cohort from Norway. We first examined the microbiota using both community- and genus-level statistical methods [31], and next explored the association between oral bacteria exhibiting pro-inflammatory hexa- or the less inflammatory penta-acylated LPS activity in relation to F_eNO_ measurements.

## Methods

### Study population

A total of 477 adult participants, generation 3 (G3), from the community-based generation study Respiratory Health In Northern Europe, Spain, and Australia (RHINESSA) [32], Bergen center, were examined in 2014–2015 with questionaries, interviews, and a clinical investigation, including gingival fluid sampling.

### Measurement of F_eNO_

F_eNO_ was measured according to standardized methods using the NIOX Mino (Aerocrine AB, Solna, Sweden), a hand-held electrochemical device, in parts per billion (ppb). Participants were instructed to refrain from exercising, smoking, eating, and drinking for at least 1 h before measurement, according to the guidelines [33]. During the procedure, participants were seated in an upright position wearing a nose clip to allow them to inhale NO-free air through the device and subsequently exhale it at a flow rate of 50 ml/s for 10 s, which mainly reflects NO production from the central airways [33, 34]. The participants were divided into three F_eNO_ categories: F_eNO_ < 25 ppb used as a reference, 25–49 ppb, and ≥ 50 ppb, corresponding to low, intermediate, and high F_eNO_ levels, respectively, according to the American Thoracic Society (ATS) guidelines on the interpretation of F_eNO_ for clinical applications [35].

### Gingival fluid sampling

Gingival fluid was collected with sterile paper points (PROTAPER, Jacobsen Dental, Norway) from the gingival crevice (area between the gingiva crest and the neck of the tooth) at five per-protocol predetermined sites in the lower and upper jaw, specifically between the left front tooth (lateral side), the right front tooth (lateral side), left and right molars number 6 (both facing molar 5). The paper points were collected into 2 ml Biopur Safe-Lock tubes without buffer and stored at − 80 °C until further processing; five paper points per individual from the upper or lower jaw were stored in each tube.

### DNA extraction, amplification, Illumina MiSeq sequencing, and data preprocessing

Bacterial DNA was extracted from the gingival fluid samples and based on all five paper points from the lower jaw. A total of 12.5 ng DNA was amplified using a combination (4:1) of universal and *Bifidobacterium*-specific primers targeting the V1-V2 region of the 16S rRNA gene [36, 37]; primer sequences contained overhang adapters appended to the 5’ end of each primer for compatibility with Illumina sequencing platform. The complete sequences of the primers were:

8F (5ʹ TCGTCGGCAGCGTCAGATGTGTATAAGAGACAGAGAGTTTGATCCTGGCTCAG 3ʹ), BifidoF (5ʹ TCGTCGGCAGCGTCAGATGTGTATAAGAGACAGAGGGTTCGATTCTGGCTCAG 3ʹ), 338R (5ʹ GTCTCGTGGGCTCGGAGATGTGTATAAGAGACAGGCTGCCTCCCGTAGGAGT 3ʹ).

Master mixes contained 12.5 ng of total DNA, 0.2 μM of each primer, and 2 × KAPA HiFi HotStart ReadyMix (KAPA Biosystems, Wilmington, MA). The amplification program was as follows: 3 min at 95 °C, followed by 25 cycles of 30 s at 95 °C, 30 s at 55 °C and 30 s at 72 °C, with an extension step at 72 °C for 5 min and a final hold at 4 °C. The 16S amplicons were purified using the AMPure XP reagent (Beckman Coulter, Indianapolis, IN). Illumina sequencing adapters and dual-index barcodes (index 1(i7) and index 2(i5)) (Illumina, San Diego, CA) were added to the amplicons using a limited-cycle PCR program: 3 min at 95 °C, followed by 8 cycles of 30 s at 95 °C, 30 s at 55 °C and 30 s at 72 °C, with an extension step at 72 °C for 5 min and a final hold at 4 °C. The resulting libraries were again purified using the AMPure XP reagent (Beckman Coulter, Indianapolis, IN), quantified, and normalized prior to pooling. The DNA library pool was then denatured with NaOH, diluted with hybridization buffer, and heat-denatured before loading on the MiSeq reagent cartridge (Illumina) and on the MiSeq instrument (Illumina, San Diego, CA). Automated cluster generation and paired-end MiSeq sequencing were performed at the UNC Microbiome Core Facility at the University of North Carolina, USA according to the manufacturer’s instructions.

Illumina MiSeq sequencing output was converted to fastq-format and demultiplexed using bcl2fastq v.2.18.0.12; the resulting paired-end reads were processed in QIIME 2 v. 2018.11 [38]. Barcodes and linker primer sequences were trimmed using the QIIME 2 invocation of cutadapt [39]. DADA2 [40] was used for denoising and dereplicating of paired-end reads, quality filtering, error correction, and chimera detection using default parameters. Amplicon sequence variants (ASVs; mean length 308.23 (± 28.17) nt) from DADA2 were assigned taxonomy against the Human Oral Microbiome Database v.15.1 (HOMD; http://www.homd.org) [41] using the *q2-feature-classifier* plugin with a setting *classify-sklearn* [42]. De novo multiple sequence alignment was generated with MAFFT [43] and phylogenetically uninformative or ambiguously aligned columns were removed prior to constructing a rooted phylogenetic tree in FastTree2 [44] (QIIME 2 plugins *alignment mafft*, *alignment mask*, *phylogeny fasttree*, and *phylogeny midpoint-root*, respectively).

### Lipid A annotation

Annotation of oral bacteria to hexa- or penta-acylated LPS producers was done according to Brix et al. [14]; it was based on the presence/absence of the nine genes in the Raetz pathway in the whole genome sequenced bacteria. Bacteria containing all nine genes, including *LpxL* and *LpxM*, were assigned to have the ability to produce hexa-acylated LPS. Bacteria with the eight first genes in the Raetz pathway, and not *LpxM*, were annotated to have the ability for penta-acylated LPS production.

### Biostatistics: associations between oral microbiota and F_eNO_

Descriptive statistics are presented as mean [± standard deviation (sd)] and median [interquartile range (IQR)] for continuous variables (compared by Shapiro–Wilk test) and as frequency (percentage) for categorical variables. Potential confounders were selected based on the factors that we know affect both the exposure (oral microbiota composition) and the outcome (F_eNO_) from the RHINESSA cohort [32] using directed acyclic graphs (DAGs). We adjusted for age, sex, height, weight, smoking habits (never/previous/current), gingival bleeding upon brushing teeth (never/rarely/sometimes/often/always), an attack of asthma in the last 12 months (no/yes), and the use of asthma medication (no/yes). Participants who had used antibiotics in the last 4 weeks before gingival sampling were excluded from the downstream analyses [45].

We applied community-level analysis and differential abundance analysis. At the community level, the microbiome regression-based kernel association test (MiRKAT) [46] was used to test for associations between F_eNO_ and differential composition of the overall, only hexa-, or only penta-acylated LPS-producing oral bacteria. As compared to a traditional permutation-based distance analysis PERMANOVA [47], MiRKAT is able to consider multiple distance metrics by constructing corresponding kernel matrices while controlling for possible confounding effects. In our study, four kernels were tested simultaneously with an omnibus test (via the Cauchy combination test which allows the weighting of all kernels equally) to increase the power of the analyses and to obtain more insight into the source of microbial variability [48, 49]. We used four different beta diversity metrics: unweighted UniFrac, weighted UniFrac, generalized UniFrac, and Bray–Curtis dissimilarity. The abundance-based Bray–Curtis dissimilarity relies solely on the abundances of bacterial taxa between two different samples [50]. In contrast, the UniFrac distances differ by the weighting of phylogeny upon measuring similarity between samples [51]. Unweighted UniFrac is defined as a binary, or presence/absence, weighting, and it is most efficient in detecting abundance change in rare taxa given that the abundant taxa are likely to be present in all samples [52]. Weighted UniFrac uses taxon abundance information and has more power to detect changes in common taxa [53]. The generalized UniFrac moderates the weighting placed on either abundant or rare taxa and therefore has more power to detect changes in bacterial species with modest abundance [54]; it can be done by tuning an additional parameter α that we set to 0.5.

Function *GUniFrac* from the GUniFrac R package was used to generate the UniFrac distances [54]. We rarefied the data before calculating beta diversity metrics to account for variability in sequencing depth (function *rrarefy* from the vegan R package) [55, 56]. Beta diversities on non-rarefied data were also provided and served as a sensitivity analysis. The kernel-specific p-values were computed by inverting the characteristic function of the mixture chi-square distribution (method = “*davies*”) [57]. We adjusted for age, sex, height, weight, smoking habits, gum bleeding, an attack of asthma in the last 12 months, and the use of asthma medication. Samples with missing metadata on one or more covariates were removed prior to running the MiRKAT.

Analysis of compositions of microbiomes with bias correction (ANCOM-BC) [58] was used to test which bacterial taxa were differentially abundant between three clinically defined F_eNO_ categories (F_eNO_ < 25 ppb used as a reference group) while controlling for other covariates (see above). ANCOM-BC accounts for the compositional nature of microbiome data [59], reports direction and effect size for absolute abundances, and allows multiple testing [58]. The tool provides a statistically valid test with a q-value (adjusted p-value) and confidence intervals (log fold change) for each bacterial taxon. A negative and a positive log fold change indicate that the taxon is less or more abundant, respectively, compared to the reference group. All p-values were adjusted with the Holm-Bonferroni method which is a default option in ANCOM-BC; it is recommended when interactions between taxa are unknown. Significance was assessed with a threshold of false discovery rate (fdr) < 0.05. The other settings for implementing *ancombc* function were as follows: *prv_cut* = 0.10; *lib_cut* = 1000; *struc_zero* = TRUE; *neg_lb* = TRUE; *tol* = 1e−5, *max_iter* = 100, *conserve* = FALSE, *global* = TRUE. Prior to running the ANCOM-BC, we aggregated bacterial ASVs into genera (function *aggregate_taxa*, microbiome R package).

Since current smoking is associated with lower F_eNO_ levels [60, 61] and alters oral bacterial community composition [62], and individuals with asthma are usually characterized by increased F_eNO_ [63], we performed two sensitivity analyses. The definition of current asthma was based on the affirmative answers to the questions about having had an attack of asthma in the last 12 months and/or currently taking asthma medication. We excluded (1) current smokers (and did not adjust for smoking habits in the MiRKAT and ANCOM-BC models), and (2) subjects with current asthma (and did not adjust for an attack of asthma in the last 12 months and the use of asthma medication in the MiRKAT and ANCOM-BC models).

All statistical analyses were performed in R version 4.2.2 [64] using packages ANCOMBC v.1.6.2 [58], ggplot2 v.3.3.6 [65], GUniFrac v.1.6 [66], microbiome v.1.18.0 [67], MiRKAT v.1.2.1 [46], phyloseq v.1.40.0 [68], tidyverse v.1.3.2 [69] and vegan v.2.6-2 [70].

## Results

### Study population characteristics in relation to F_eNO_

A total of 477 adults from the community-based generation study RHINESSA, Bergen center, were analyzed, 223 (47%) women and 254 (53%) men. The women were slightly younger than the men (mean (± sd): 27.2 (± 6.6) and 28.7 (± 6.8) years; median (IQR): 26 (22–32) and 28 (23–34) years; min–max: 18–45 and 18–47 years, respectively). The outcome variable F_eNO_ had a right-skewed distribution, with median (IQR) equal to 18 (13–25) ppb. The range of F_eNO_ values was 5–158 ppb for the total population, 5–123, and 5–158 ppb for the women and men, respectively. When the participants were divided into three F_eNO_ categories, the distribution was as follows: 345 (72.9%), 113 (23.9%), and 15 (3.2%) corresponding to low, intermediate, and high F_eNO_ levels, respectively (Table 1). More women than men had low vs. high F_eNO_ levels (186 (53.9%) vs. 4 (26.7%) women, and 159 (46.1%) vs. 11 (73.3%) men, respectively), excluding four participants with missing information on F_eNO_ (Table 1). Most current smokers (*n* = 56) had low F_eNO_ values, while only 2 participants had F_eNO_ ≥ 50 ppb (both males) (Table 1). The number of individuals with current asthma was 30 (6.3% of the population). When these individuals were subdivided into low, intermediate, and high F_eNO_ categories, the distribution was 20 (5.8%), 7 (6.2%), and 3 (20.0%) individuals, respectively. Due to the small number of subjects with current asthma in our study population, we adjusted for an attack of asthma in the last 12 months and the use of asthma medication separately in the models.

**Table 1:** Characteristics of the study population in relation to F_eNO_ categories

Variable	F_eNO_ group		
	Low F_eNO_	Intermediate F_eNO_	High F_eNO_
	(< 25 ppb^a^)	(25–49 ppb)	(≥ 50 ppb)
Individuals, *n* (%)			
	345 (72.9)	113 (23.9)	15 (3.2)
Sex, *n* (%)			
Female^b^	186 (53.9)	30 (26.5)	4 (26.7)
Male^b^	159 (46.1)	83 (73.5)	11 (73.3)
Smoking habits, *n* (%)			
Never^c^	238 (69.0)	82 (72.6)	12 (80.0)
Previous	51 (14.8)	24 (21.2)	1 (6.7)
Current	56 (16.2)	7 (6.2)	2 (13.3)
Current asthma, *n* (%)			
Yes	20 (5.8)	7 (6.2)	3 (20.0)

^a^*ppb* parts per billion

^b^Participants with missing information on F_eNO_: female (*n* = 3) and male (*n* = 1)

^c^Never and Current smoker were defined as having answered no and yes, respectively, to the question “Do you smoke?”. Previous smoker was defined as having answered yes to the question “Did you smoke previously?”

### Bacterial diversity and taxa distribution

A total of 47,443,921 reads representing 33,756 ASVs were obtained from the gingival samples of the 477 subjects (an average number of reads per sample: 99,463; range: 10,017–292,207).

The dominant bacterial phyla were Firmicutes (27.7%), Bacteroidetes (24.7%), Fusobacteria (18.4%), Proteobacteria (15.6%), and Actinobacteria (8.5%). The most prevalent bacterial genera, present in all samples, were *Fusobacterium* (15.2%, phylum Fusobacteria), *Streptococcus* (9.7%, phylum Firmicutes), and *Prevotella* (8.4%, phylum Bacteroidetes).

### Lipid A annotation

**Fig. 1:**
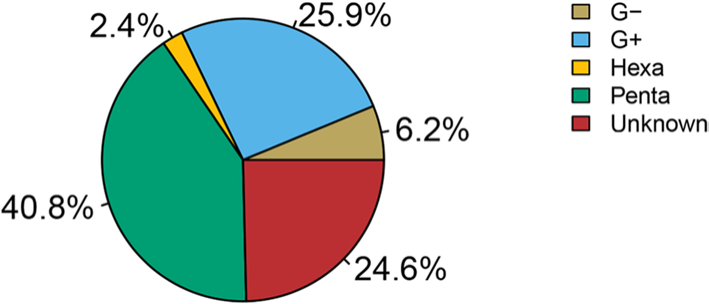
The distribution of the oral microbiota annotated to hexa- and penta-acylated LPS producers, as well as Gram-negative (G−) that were not possible to assign to hexa- or penta-acylated LPS groups due to phylum annotation only, Gram-positive (G +), and unknown bacteria from the study population

In total, 2.4% and 40.8% of the oral bacterial genera were annotated as hexa- and penta-acylated LPS producers, respectively (Fig. 1). Among pro-inflammation-inducing oral bacteria, 9 genera from the phylum Proteobacteria were detected (*Aggregatibacter*, *Enterobacter*, *Escherichia*, *Haemophilus*, *Klebsiella*, *Proteus*, *Stenotrophomonas*, *Yersinia*, and unclassified Proteobacteria). Penta-acylated LPS producers were represented by 71 genera, with the most abundant belonging to *Capnocytophaga* and *Prevotella* (phylum Bacteroidetes), *Neisseria* (phylum Proteobacteria), and *Fusobacterium* (phylum Fusobacteria).

### Community-level analyses with MiRKAT

We investigated the association between the oral microbiota community composition at the overall level, and for the hexa-, and penta-acylated LPS producers vs. F_eNO_ as the marker of airway inflammation, while controlling for potential confounders. Before the analyses, we filtered out 35 samples (7.3%) with missing data on one or more covariates. We performed the analysis on both rarefied and non-rarefied data to show that the variability in sequencing depth does not affect the results. The kernel-specific and omnibus p-values for each of the analyzed bacterial community compositions and the model parameters are shown in Table 2 and Additional file 1: Table S1. Taking into account the relative relatedness of community members (the UniFrac metrics), the association between the overall bacterial community composition and F_eNO_ was statistically significant (the omnibus p < 0.05). But the same effect was not observed between F_eNO_ and the overall oral bacterial community of hexa- or penta-acylated LPS producers. On the contrary, we identified a statistically significant relation between F_eNO_ and the Bray–Curtis kernel for both hexa- and penta-acylated LPS community compositions, while this was not observed for the overall bacterial community (Table 2 and Additional file 1: Table S1). These results imply that the relatedness between the hexa- and penta-acylated LPS producers, with hexa-acylated LPS producers being Gammaproteobacteria and penta-acylated LPS producers being dominated by the highly abundant Bacteroidetes members, may explain this result, as the bacterial relatedness is taken into account for all three UniFrac metrics, but not for Bray–Curtis dissimilarity.

**Table 2:** Kernel-specific and omnibus p-values for each of the analyzed bacterial community compositions (rarefied data) vs. F_eNO_ (a continuous variable)

K_U_	K_W_	K_G_	K_BC_	Omnibus p-value
Overall community composition				
**0.005**	**0.005**	**0.015**	0.159	**0.008**
Hexa-acylated LPS community composition				
0.099	0.150	0.120	**0.040**	0.081
Penta-acylated LPS community composition				
0.144	0.174	0.159	**0.024**	0.066

We adjusted for age, sex, height, weight, smoking habits, gum bleeding, the use of asthma medication, and an attack of asthma in the last 12 months. K_U_, K_W_, K_G_, and K_BC_ represent MiRKAT results for the unweighted UniFrac kernel, weighted UniFrac kernel, generalized UniFrac kernel with α = 0.5, and Bray–Curtis kernel, respectively. The omnibus p-value is computed via the Cauchy combination test which allows the weighting of all four kernels equally. Both the kernel-specific and omnibus p-values were obtained by the Davies method. Significance < 0.05 is shown in bold

We also performed two sensitivity analyses excluding (1) current smokers, and (2) subjects with current asthma (Additional file 1: Tables S2 and S3). The associations between the overall bacterial community composition and F_eNO_ were not statistically significant when current smokers and subjects with current asthma were excluded. In other words, in healthy, non-asthmatic subjects the contribution of oral bacteria to the production of nitric oxide can be attributed to inter-individual variation.

### Differential abundance testing with ANCOM-BC

ANCOM-BC identified 27 differentially abundant bacterial genera from the phyla Firmicutes (12), Bacteroidetes (2), Proteobacteria (8), Actinobacteria (4), and Saccharibacteria (1) that significantly differed between the individuals with high vs. low F_eNO_ levels while adjusting for confounders (Table 3). Of note is that the significances of these genera are due to structural zeros [71] and they were detected by the presence/absence test. Among those taxa, 11 bacterial genera are potential penta-acylated LPS producers (Table 3). No hexa-acylated LPS-producing oral bacteria were differentially abundant between the groups.

**Table 3:** Differentially abundant bacterial genera between the participants with low F_eNO_ vs. high F_eNO_ levels detected by ANCOM-BC (*n* = 477)

Genus	Phylum	Low F_eNO_	Intermediate F_eNO_	High F_eNO_	Lipid A annotation
		(< 25 ppb^a^)	(25–49 ppb)	(≥ 50 ppb)	
*Achromobacter*	Proteobacteria	0.81	0.19	0.00	Penta
*Aerococcus*	Firmicutes	0.92	0.08	0.00	Gram-positive
*Agrobacterium*	Proteobacteria	0.74	0.25	0.01	Penta
*Bacteroides*	Bacteroidetes	0.73	0.26	0.02	Penta
*Bosea*	Proteobacteria	0.87	0.12	0.00	Penta
*Brevundimonas*	Proteobacteria	0.68	0.32	0.00	Penta
*Clostridiales_[F-1][G-2]*	Firmicutes	0.97	0.03	0.00	Gram-positive
*Clostridiales_[F-3][G-1]*	Firmicutes	0.86	0.14	0.00	Gram-positive
*Cutibacterium*	Actinobacteria	0.80	0.20	0.01	Gram-positive
*Dermabacter*	Actinobacteria	0.65	0.33	0.02	Gram-positive
*Erysipelothrix*	Firmicutes	0.82	0.17	0.01	Gram-positive
*Helicobacter*	Proteobacteria	0.81	0.17	0.02	Penta
*Janibacter*	Actinobacteria	0.79	0.20	0.01	Gram-positive
*Kocuria*	Actinobacteria	0.77	0.22	0.00	Gram-positive
*Lachnospiraceae_[G-7]*	Firmicutes	0.81	0.19	0.00	Gram-positive
*Lactobacillus*	Firmicutes	0.80	0.20	0.00	Gram-positive
*Lysinibacillus*	Firmicutes	0.72	0.26	0.02	Gram-positive
*Mitsuokella*	Firmicutes	0.94	0.06	0.01	Penta
*Mogibacterium*	Firmicutes	0.78	0.22	0.00	Gram-positive
*Moraxella*	Proteobacteria	0.95	0.05	0.00	Penta
*Novosphingobium*	Proteobacteria	0.68	0.31	0.00	Penta
*Paenibacillus*	Firmicutes	0.62	0.36	0.02	Gram-positive
*Pedobacter*	Bacteroidetes	0.64	0.35	0.01	Penta
*Peptostreptococcaceae_[XI][G-2]*	Firmicutes	0.85	0.14	0.01	Gram-positive
*Saccharibacteria_(TM7)_[G-4]*	Saccharibacteria	0.58	0.40	0.02	Gram-negative
*Schlegelella*	Proteobacteria	0.84	0.15	0.01	Penta
*Staphylococcus*	Firmicutes	0.82	0.17	0.01	Gram-positive

Data are presented as relative abundances for each genus per F_eNO_ category. The lipid A annotation of penta-acylated LPS producers is indicated as “Penta”

^a^*ppb* parts per billion

We also performed two sensitivity analyses excluding (1) current smokers, and (2) subjects with current asthma (Additional file 1: Tables S4 and S5). Among 25 differentially abundant bacterial genera, 11 and 8 are potential penta-acylated LPS-producing oral bacteria (Additional file 1: Tables S4 and S5). Of note is that there is a large overlap of differentially abundant taxa between the primary and sensitivity analyses (23 and 22 bacterial genera, respectively).

## Discussion

To the best of our knowledge, this study is the first to explore the association between LPS-producing oral bacteria of different potency and exhaled nitric oxide in a population-based adult cohort from Norway. We observed a strong association between F_eNO_ and the overall community composition of oral bacteria based on several ecologically informative kernels. The Bray–Curtis dissimilarity within hexa- and penta-acylated LPS producers was associated with increasing F_eNO_ levels independent of covariate adjustment. At a lower taxonomic level, 27 penta-acylated LPS-producing bacterial genera were more abundant in individuals with low F_eNO_ vs. high F_eNO_, while hexa-acylated LPS producers were found not to be enriched.

Bacterial LPS may induce various biological responses that may be beneficial or harmful for the host, and frequent exposure to low levels of LPS early in life is essential for the development of a healthy immune system [16, 21, 72, 73]. In healthy individuals, Gram-negative bacteria and their LPS (10 ± 20 pg/mL) have been detected in the lower intestine, saliva, dental plaque, skin, lungs, respiratory and urinary tracts [3]. The LPS biosynthesis is highly conserved in Gram-negative bacteria, but there are exceptions [5, 74]. Strongly agonistic LPS is comprised of a rather similar set of lipid A types [1]. For example, members of Proteobacteria, class Gammaproteobacteria (e.g. genera *Enterobacter*, *Escherichia*, *Klebsiella*), with a few exceptions, express hexa-acylated lipid A forms, whereas Bacteroidetes species (e.g. *Bacteroides* and *Prevotella*) are known to produce penta- and tetra-acylated lipid As that elicit reduced TLR4 responses [3, 16]. Contrarily, members of Actinobacteria and Firmicutes are Gram-positive and generally do not produce LPS [75]. In the oral cavity of our study population, Bacteroidetes was the second most abundant phylum compared to Proteobacteria (24.7% and 15.6%, respectively). Brix et al. [14] reported that the lung microbiota of healthy individuals present a low ratio of hexa- to penta-acylated LPS producers compared to the lung microbiota of individuals with asthma. We speculate that the pro-inflammatory effect of hexa-acylated LPS-producing oral bacteria may be counteracted by the dominance of the more abundant penta-acylated LPS producers, such as Bacteroidetes species, in this population-based adult cohort involving mainly healthy individuals. In a healthy gut, the prevalence of Proteobacteria and Bacteroidetes species and structural and functional differences between their lipid A forms were shown to be crucial for the maintenance of gut homeostasis [3, 16]. Moreover, most lipid A forms produced in the gut inhibit the immune response, with only a small fraction of them functioning as a TLR4-agonist [16, 76]. It implies that bacterial LPS has the potential to modulate host tolerance and influence the outcome of microbiota-linked diseases [76] and that the composition of bacterial communities, including their metabolites, acts as a mediator of inflammation [77].

To explore the association between the oral microbiota composition and F_eNO_, we applied the community- (MiRKAT) and genus-level (ANCOM-BC) statistical methods. In MiRKAT, the choice of a distance matrix to generate an ecologically informative kernel strongly affects the statistical power [46, 48]. The use of multiple beta-diversity metrics with an omnibus test increases the power of analyses and allows distinct distances to capture distinct association patterns [48, 51]. Weighted UniFrac relies more heavily on the deep structure of the phylogeny, whereas unweighted UniFrac and abundance-based metrics rely more heavily on the shallow branches in the phylogeny [51]. In our study, the overall oral bacterial community composition measured via kernels obtained by transforming weighted and generalized UniFrac metrics was significantly associated with F_eNO_; contrarily, the association between the community compositions of hexa- or penta-acylated LPS producers and F_eNO_ was significant only when Bray–Curtis dissimilarity was used as a kernel (Table 2). In addition, only penta-acylated LPS producers (11 distinct genera, Table 3) were more abundant in individuals with low vs. high F_eNO_, while hexa-acylated LPS producers were found not to be enriched. Based on our finding of a 20-fold higher abundance of penta-acylated LPS-producing bacterial genera in the oral cavity of this cohort, we speculate that most of the oral LPS of adults from the general population, similar to the ones from the healthy human gut and lung, is immunoinhibitory [14, 16, 76].

The strength of our study is that the findings are based on a well-characterized, homogeneous adult cohort from the general population with a relatively large sample size (*n* = 477). One of the limitations of our study is the insufficient resolution of the 16S rRNA gene in delineating ASVs below genus level and unknown but substantial strain-level diversity which might be unique to some individuals [78]. Moreover, amplicon sequencing provides detailed taxonomic profiling of bacterial communities but not their functional potential [79]. In addition, factors not measured in the present study, e.g. diet, physical activity, respiratory infections, air pollution, and allergen exposure, can positively or negatively affect F_eNO_ levels [35, 61, 80], and therefore their effect on the association of the oral bacterial community composition and F_eNO_ remains unknown. Although the measurement of F_eNO_ is a rapid and non-invasive procedure, it reflects only some, although not all, aspects of airway inflammation [81, 82]. Thus, the results presented here should be interpreted with caution.

## Conclusions

The oral cavity is an open, heterogeneous, and highly dynamic environment with distinct bacterial community composition and complex species interactions in both health and disease. To the best of our knowledge, this study is the first to provide evidence that the composition of different lipid A variants in the oral cavity of healthy subjects may determine the level of immune activation triggered by microbiota-derived endotoxins. It is likely that the pro-inflammatory effect of hexa-acylated LPS producers is counteracted by the dominance of the penta-acylated LPS producers which may reduce inflammatory processes. Even though the pro-inflammatory oral bacteria were present in lower abundances, they may reach the lungs through micro-aspiration or systemic dispersal, e.g. injuries during toothbrushing, flossing, or mastication, and thereby indirectly contribute to airway inflammation.

## Supplementary Information

**Additional file 1: Table S1. **Kernel-specific and omnibus p-values for each of the analyzed bacterial community compositions (non-rarefied data) vs. F_eNO_ (a continuous variable). We adjusted for age, sex, height, weight, smoking habits, gum bleeding, the use of asthma medication, and an attack of asthma in the last 12 months. **Table S2. **Kernel-specific and omnibus p-values for the overall bacterial community composition (rarefied data; 411 participants, current smokers excluded) vs. F_eNO_ (a continuous variable). We adjusted for age, sex, height, weight, gum bleeding, the use of asthma medication, and an attack of asthma in the last 12 months. **Table S3. **Kernel-specific and omnibus p-values for the overall bacterial community composition (rarefied data; 445 participants, subjects with current asthma excluded) vs. F_eNO_ (a continuous variable). We adjusted for age, sex, height, weight, smoking habits and gum bleeding. **Table S4. **Differentially abundant bacterial genera between the participants with low F_eNO_ vs. high F_eNO_ levels detected by ANCOM-BC (*n *= 411, current smokers excluded). Data are presented as relative abundances for each genus per F_eNO_ category. The lipid A annotation of penta-acylated LPS producers is indicated as “Penta”. **Table S5. **Differentially abundant bacterial genera between the participants with low F_eNO_ vs. high F_eNO_ levels detected by ANCOM-BC (*n *= 445, subjects with current asthma excluded). Data are presented as relative abundances for each genus per F_eNO_ category. The lipid A annotation of penta-acylated LPS producers is indicated as “Penta”.

## Data Availability

Amplicon sequencing data analyzed during the current study are available in the Dryad repository (https://doi.org/10.5061/dryad.r2280gbfh).

## Abbreviations

ANCOM-BC: Analysis of compositions of microbiomes with bias correction
fdr: False discovery rate
F_eNO_: Fractional exhaled nitric oxide
IQR: Interquartile range
MiRKAT: Microbiome regression-based kernel association test
nt: Nucleotide
NO: Nitric oxide
ppb: Parts per billion
sd: Standard deviation
