# Supplementary material for: Association between lipid-A-producing oral bacteria of different potency and fractional exhaled nitric oxide in a Norwegian population-based adult cohort
Source: J Transl Med. 2023 May 29;21:354. doi: 10.1186/s12967-023-04199-z (PMC10226232; doi:10.1186/s12967-023-04199-z)
Supplement: Supplementary file 1 — Additional file 1: Table S1. Kernel-specific and omnibus p-values for each of the analyzed bacterial community compositions (non-rarefied data) vs. FeNO (a continuous variable). We adjusted for age, sex, height, weight, smoking habits, gum bleeding, the use of asthma medication, and an attack of asthma in the last 12 months. Table S2. Kernel-specific and omnibus p-values for the overall bacterial community composition (rarefied data; 411 participants, current smokers excluded) vs. FeNO (a continuous variable). We adjusted for age, sex, height, weight, gum bleeding, the use of asthma medication, and an attack of asthma in the last 12 months. Table S3. Kernel-specific and omnibus p-values for the overall bacterial community composition (rarefied data; 445 participants, subjects with current asthma excluded) vs. FeNO (a continuous variable). We adjusted for age, sex, height, weight, smoking habits and gum bleeding. Table S4. Differentially abundant bacterial genera between the participants with low FeNO vs. high FeNO levels detected by ANCOM-BC (n = 411, current smokers excluded). Data are presented as relative abundances for each genus per FeNO category. The lipid A annotation of penta-acylated LPS producers is indicated as “Penta”. Table S5. Differentially abundant bacterial genera between the participants with low FeNO vs. high FeNO levels detected by ANCOM-BC (n = 445, subjects with current asthma excluded). Data are presented as relative abundances for each genus per FeNO category. The lipid A annotation of penta-acylated LPS producers is indicated as “Penta”. [file 12967_2023_4199_MOESM1_ESM.pdf]

Association between lipid-A-producing oral bacteria of different potency and fractional exhaled nitric oxide in a Norwegian population-based adult cohort

Maryia Khomich[1*], Huang Lin[2], Andrei Malinovschi[3], Susanne Brix[4], Lucia Cestelli[1], Shyamal Peddada[2], Ane Johannessen[5], Carsten Eriksen[4,6], Francisco Gomez Real[1,7], Cecilie Svanes[5,8], Randi Jacobsen Bertelsen[1,9*]

[1]Department of Clinical Science, University of Bergen, Bergen, Norway

[2]Biostatistics and Computational Biology Branch, National Institute of Environmental Health Sciences (NIEHS), NIH, Research Triangle Park, NC, USA

[3]Department of Medical Sciences, Clinical Physiology, Uppsala University, Uppsala, Sweden

[4]Department of Biotechnology and Biomedicine, Technical University of Denmark, Kongens Lyngby, Denmark

[5]Department of Global Public Health and Primary Care, Center for International Health, University of Bergen, Bergen, Norway

[6]Center for Molecular Prediction of Inflammatory Bowel Disease (PREDICT), Department of Clinical Medicine, Aalborg University, Copenhagen, Denmark

[7]Department of Obstetrics and Gynecology, Haukeland University Hospital, Bergen, Norway

[8]Department of Occupational Medicine, Haukeland University Hospital, Bergen, Norway

[9]Oral Health Center of Expertise in Western Norway, Bergen, Norway

*Corresponding authors:

Maryia Khomich, maryia.khomich@uib.no, ORCID: 0000-0002-6840-5739

Randi Jacobsen Bertelsen, randi.j.bertelsen@uib.no, ORCID: 0000-0001-5319-525X

Additional file Tables S1-S5

Table S1. Kernel-specific and omnibus p-values for each of the analyzed bacterial community compositions (non-rarefied data) vs. F_{eNO} (a continuous variable). We adjusted for age, sex, height, weight, smoking habits, gum bleeding, the use of asthma medication, and an attack of asthma in the last 12 months.

K_U	K_W	K_G	K_{BC}	Omnibus p-value
Overall community composition				
0.063	**0.005**	**0.014**	0.183	**0.014**
Hexa-acylated LPS community composition				
0.771	0.275	0.268	**0.013**	0.050
Penta-acylated LPS community composition				
0.186	0.189	0.180	**0.028**	0.078

K_U, K_W, K_G, and K_{BC} represent MiRKAT results for the unweighted UniFrac kernel, weighted UniFrac kernel, generalized UniFrac kernel with α = 0.5, and Bray-Curtis kernel, respectively. The omnibus p-value is computed via the Cauchy combination test which allows the weighting of all four kernels equally. Both the kernel-specific and omnibus p-values were obtained by the Davies method. Significance < 0.05 is shown in bold.

Table S2. Kernel-specific and omnibus p-values for the overall bacterial community composition (rarefied data; 411 participants, current smokers excluded) vs. F_{eNO} (a continuous variable). We adjusted for age, sex, height, weight, gum bleeding, the use of asthma medication, and an attack of asthma in the last 12 months.

K_U	K_W	K_G	K_{BC}	Omnibus p-value
0.416	0.327	0.200	0.570	0.351

K_U, K_W, K_G, and K_{BC} represent MiRKAT results for the unweighted UniFrac kernel, weighted UniFrac kernel, generalized UniFrac kernel with $\alpha = 0.5$, and Bray-Curtis kernel, respectively. The omnibus p-value is computed via the Cauchy combination test which allows the weighting of all four kernels equally. Both the kernel-specific and omnibus p-values were obtained by the Davies method. Significance < 0.05.

Table S3. Kernel-specific and omnibus p-values for the overall bacterial community composition (rarefied data; 445 participants, subjects with current asthma excluded) vs. F_{eNO} (a continuous variable). We adjusted for age, sex, height, weight, smoking habits and gum bleeding.

K_U	K_W	K_G	K_{BC}	Omnibus p-value
0.751	0.717	0.895	0.738	0.804

K_U, K_W, K_G, and K_{BC} represent MiRKAT results for the unweighted UniFrac kernel, weighted UniFrac kernel, generalized UniFrac kernel with $\alpha = 0.5$, and Bray-Curtis kernel, respectively. The omnibus p-value is computed via the Cauchy combination test which allows the weighting of all four kernels equally. Both the kernel-specific and omnibus p-values were obtained by the Davies method. Significance < 0.05.

Table S4. Differentially abundant bacterial genera between the participants with low F_{eNO} vs. high F_{eNO} levels detected by ANCOM-BC (*n* = 411, current smokers excluded). Data are presented as relative abundances for each genus per F_{eNO} category. The lipid A annotation of penta-acylated LPS producers is indicated as "Penta".

**Genus**	**Phylum**	**Low F_{eNO} (< 25 ppb*)**	**Intermediate F_{eNO} (25-49 ppb)**	**High F_{eNO} (≥ 50 ppb)**	**Lipid A annotation**
Aerococcus	Firmicutes	0.92	0.08	0.00	Gram-positive
Agrobacterium	Proteobacteria	0.74	0.26	0.00	Penta
Bacteroides	Bacteroidetes	0.71	0.28	0.01	Penta
Bosea	Proteobacteria	0.88	0.12	0.00	Penta
Brevundimonas	Proteobacteria	0.61	0.39	0.00	Penta
Clostridiales_[F-1][G-2]	Firmicutes	0.96	0.03	0.00	Gram-positive
Clostridiales_[F-3][G-1]	Firmicutes	0.85	0.15	0.00	Gram-positive
Dermabacter	Actinobacteria	0.60	0.38	0.02	Gram-positive
Erysipelothrix	Firmicutes	0.81	0.18	0.00	Gram-positive
Flavitalea	Bacteroidetes	0.75	0.24	0.01	Penta
Helicobacter	Proteobacteria	0.77	0.21	0.02	Penta
Janibacter	Actinobacteria	0.76	0.24	0.00	Gram-positive
Lachnospiraceae_[G-7]	Firmicutes	0.75	0.25	0.01	Gram-positive
Lactobacillus	Firmicutes	0.78	0.22	0.00	Gram-positive
Microbacterium	Actinobacteria	0.73	0.27	0.00	Gram-positive
Mitsuokella	Firmicutes	0.94	0.06	0.00	Penta
Mogibacterium	Firmicutes	0.75	0.25	0.00	Gram-positive
Moraxella	Proteobacteria	0.96	0.04	0.00	Penta

**Genus**	**Phylum**	**Low F_{eNO} (< 25 ppb*)**	**Intermediate F_{eNO} (25-49 ppb)**	**High F_{eNO} (≥ 50 ppb)**	**Lipid A annotation**
Novosphingobium	Proteobacteria	0.64	0.35	0.00	Penta
Paenibacillus	Firmicutes	0.62	0.37	0.00	Gram-positive
Pedobacter	Bacteroidetes	0.59	0.41	0.00	Penta
Peptostreptococcaceae_[XI][G-2]	Firmicutes	0.82	0.17	0.01	Gram-positive
Saccharibacteria_(TM7)_[G-4]	Saccharibacteria	0.56	0.42	0.02	Gram-negative
Schlegelella	Proteobacteria	0.76	0.22	0.02	Penta
Staphylococcus	Firmicutes	0.72	0.27	0.01	Gram-positive

*ppb: parts per billion.

Table S5. Differentially abundant bacterial genera between the participants with low F_{eNO} vs. high F_{eNO} levels detected by ANCOM-BC (*n* = 445, subjects with current asthma excluded). Data are presented as relative abundances for each genus per F_{eNO} category. The lipid A annotation of penta-acylated LPS producers is indicated as "Penta".

Genus	Phylum	Low F_{eNO} (< 25 ppb*)	Intermediate F_{eNO} (25-49 ppb)	High F_{eNO} (≥ 50 ppb)	Lipid A annotation
Achromobacter	Proteobacteria	0.81	0.19	0.00	Penta
Aerococcus	Firmicutes	0.93	0.07	0.00	Gram-positive
Agrobacterium	Proteobacteria	0.74	0.25	0.00	Penta
Bacteroides	Bacteroidetes	0.71	0.27	0.01	Penta
Brevundimonas	Proteobacteria	0.68	0.32	0.00	Penta
Clostridiales_[F-1][G-2]	Firmicutes	0.94	0.05	0.00	Gram-positive
Clostridiales_[F-3][G-1]	Firmicutes	0.80	0.20	0.00	Gram-positive
Cutibacterium	Actinobacteria	0.79	0.21	0.00	Gram-positive
Dermabacter	Actinobacteria	0.66	0.33	0.02	Gram-positive
Erysipelotrichaceae_[G-1]	Firmicutes	0.81	0.17	0.02	Gram-positive
Janibacter	Actinobacteria	0.79	0.20	0.01	Gram-positive
Lachnospiraceae_[G-7]	Firmicutes	0.82	0.18	0.00	Gram-positive
Lactobacillus	Firmicutes	0.80	0.20	0.00	Gram-positive
Lysinibacillus	Firmicutes	0.72	0.27	0.01	Gram-positive
Mogibacterium	Firmicutes	0.78	0.22	0.00	Gram-positive
Moraxella	Proteobacteria	0.96	0.04	0.00	Penta
Novosphingobium	Proteobacteria	0.68	0.32	0.00	Penta
Paenibacillus	Firmicutes	0.61	0.37	0.02	Gram-positive

Genus	Phylum	Low F_{eNO} (< 25 ppb*)	Intermediate F_{eNO} (25-49 ppb)	High F_{eNO} (≥ 50 ppb)	Lipid A annotation
Pedobacter	Bacteroidetes	0.63	0.36	0.01	Penta
Peptostreptococcaceae_[XI][G-2]	Firmicutes	0.85	0.14	0.00	Gram-positive
Propionibacteriaceae_[G-2]	Actinobacteria	0.66	0.32	0.01	Gram-positive
Saccharibacteria_(TM7)_[G-4]	Saccharibacteria	0.57	0.42	0.00	Gram-negative
Scardovia	Actinobacteria	0.72	0.27	0.01	Gram-positive
Schlegelella	Proteobacteria	0.85	0.14	0.00	Penta
Staphylococcus	Firmicutes	0.82	0.17	0.00	Gram-positive

*ppb: parts per billion.
